# Supplementary material for: Role of the redox state of the Pirin-bound cofactor on interaction with the master regulators of inflammation and other pathways
Source: PLoS One. 2023 Nov 30;18(11):e0289158. doi: 10.1371/journal.pone.0289158 (PMC10688961; doi:10.1371/journal.pone.0289158)
Supplement: S1 Table — (DOCX) [file pone.0289158.s001.DOCX]

Supplementary table 1: Domains of binding partners of Pirin

| **Protein** | **Domains/ Repeats** | **Description** | **Feature type** | **Start** | **End** | **Length** | **Sources** |
| --- | --- | --- | --- | --- | --- | --- | --- |
| BCL3 | ANK 1 | Ankyrin repeat 1 | Repeat | 134 | 163 | 30 | UniProt-KB |
|  | ANK 2 | Ankyrin repeat 2 | Repeat | 171 | 200 | 30 |  |
|  | ANK 3 | Ankyrin repeat 3 | Repeat | 204 | 235 | 32 |  |
|  | ANK 4 | Ankyrin repeat 4 | Repeat | 241 | 270 | 30 |  |
|  | ANK 5 | Ankyrin repeat 5 | Repeat | 275 | 304 | 30 |  |
|  | ANK 6 | Ankyrin repeat 6 | Repeat | 308 | 337 | 30 |  |
|  | ANK 7 | Ankyrin repeat 7 | Repeat | 338 | 367 | 30 |  |
| NFIX | NfI_DNAbd_pre-N | Nuclear factor I protein pre-N-terminus | Domain | 7 | 46 | 40 | Pfam |
|  | MH1 | MAD homology 1 | Domain | 68 | 172 | 105 |  |
|  | CTF_NFI | CTF/NF-I family transcription modulation region | Domain | 213 | 502 | 290 |  |
| NFKBIA | ANK 1 | Ankyrin repeat 1 | Repeat | 73 | 103 | 31 | UniProt-KB |
|  | ANK 2 | Ankyrin repeat 2 | Repeat | 110 | 139 | 30 |  |
|  | ANK 3 | Ankyrin repeat 3 | Repeat | 143 | 172 | 30 |  |
|  | ANK 4 | Ankyrin repeat 4 | Repeat | 182 | 211 | 30 |  |
|  | ANK 5 | Ankyrin repeat 5 | Repeat | 216 | 245 | 30 |  |
| SMAD9 | MH1 | MAD homology 1 | Domain | 16 | 140 | 125 | UniProt-KB |
|  | MH2 | MAD homology 2 | Domain | 273 | 467 | 195 |  |
