# Supplementary material for: Role of the redox state of the Pirin-bound cofactor on interaction with the master regulators of inflammation and other pathways
Source: PLoS One. 2023 Nov 30;18(11):e0289158. doi: 10.1371/journal.pone.0289158 (PMC10688961; doi:10.1371/journal.pone.0289158)
Supplement: S2 Table — (DOCX) [file pone.0289158.s002.DOCX]

Supplementary table 2: eQTLs in PIR and their regulated genes across different tissues

| SNP ID | Gene Symbol | Tissue |
| --- | --- | --- |
| rs4830964 | PIR | Cells - Cultured fibroblasts, Esophagus - Muscularis, Thyroid, Artery - Tibial, Nerve - Tibial, Esophagus - Gastroesophageal Junction, Heart - Left Ventricle |
|  | VEGFD | Artery - Tibial, Nerve - Tibial, Thyroid, Artery - Aorta, Esophagus - Muscularis |
|  | CA5BP1 | Lung |
|  | FANCB | Brain - Cortex |
|  | TMEM27 | Heart - Left Ventricle, Nerve - Tibial, Skin - Sun Exposed (Lower leg) |
| rs2094 | PIR | Cells - Cultured fibroblasts, Esophagus - Muscularis, Thyroid, Artery - Tibial, Nerve - Tibial, Esophagus - Gastroesophageal Junction, Heart - Left Ventricle |
|  | VEGFD | Artery - Tibial, Nerve - Tibial, Esophagus - Muscularis, Artery - Aorta, Thyroid, Muscle - Skeletal |
|  | CA5BP1 | Lung |
|  | FANCB | Brain - Cortex |
|  | TMEM27 | Heart - Left Ventricle, Nerve - Tibial |
| rs2095 | PIR | Cells - Cultured fibroblasts, Esophagus - Muscularis, Thyroid, Nerve - Tibial, Esophagus - Gastroesophageal Junction, Artery - Tibial |
|  | VEGFD | Artery - Tibial, Nerve - Tibial, Esophagus - Muscularis |
|  | TMEM27 | Artery - Coronary, Heart - Left Ventricle |
| rs908005 | PIR | Cells - Cultured fibroblasts, Thyroid, Esophagus - Muscularis, Artery - Tibial, Nerve - Tibial, Artery - Aorta, Esophagus - Gastroesophageal Junction,Adipose - Subcutaneous, Adipose - Visceral (Omentum), Brain - Caudate (basal ganglia), Testis, Lung, Brain - Nucleus accumbens (basal ganglia), Brain - Putamen (basal ganglia), Skin - Sun Exposed (Lower leg), Skin - Not Sun Exposed (Suprapubic), Pituitary, Brain - Cerebellum, Brain - Cortex, Heart - Atrial Appendage, Brain - Cerebellar Hemisphere, Muscle - Skeletal, Esophagus - Mucosa, Heart - Left Ventricle, Brain - Anterior cingulate cortex (BA24), Brain - Hippocampus, Brain - Frontal Cortex (BA9), Brain - Hypothalamus, Brain - Substantia nigra, Colon - Sigmoid, Stomach, Prostate, Whole blood, Brain - Amygdala, Breast - Mammary Tissue, Cells - EBV-transformed lymphocytes, Artery - Coronary, Colon - Transverse, Ovary, Small Intestine - Terminal Ileum, Pancreas, Brain - Spinal cord (cervical c-1), Adrenal gland, Liver |
|  | VEGFD | Artery - Tibial, Cells - Cultured fibroblasts, Thyroid, Nerve - Tibial, Muscle - Skeletal, Esophagus - Muscularis, Brain - Cerebellum, Artery - Aorta, Brain - Putamen (basal ganglia), Skin - Sun Exposed (Lower leg), Brain - Hippocampus, Brain - Cortex, Brain - Substantia nigra, Brain - Hypothalamus, Esophagus - Mucosa, Brain - Frontal Cortex (BA9), Prostate, Testis, Brain - Anterior cingulate cortex (BA24), Brain - Caudate (basal ganglia), Brain - Cerebellar Hemisphere, Brain - Nucleus accumbens (basal ganglia), Spleen, Esophagus - Gastroesophageal Junction, Heart - Atrial Appendage, Pancreas |
|  | TMEM27 | Nerve - Tibial, Esophagus - Mucosa, Skin - Sun Exposed (Lower leg), Thyroid, Brain - Cortex, Adipose - Subcutaneous, Cells - Cultured fibroblasts, Artery - Aorta, Colon - Transverse, Skin - Not Sun Exposed (Suprapubic), Artery - Tibial, Brain - Nucleus accumbens (basal ganglia), Prostate, Artery - Coronary, Muscle - Skeletal, Brain - Cerebellum, Esophagus - Gastroesophageal Junction, Adipose - Visceral (Omentum) |
|  | CA5BP1 | Spleen |
| rs1567894 | PIR | Cells - Cultured fibroblasts, Esophagus - Muscularis, Thyroid, Artery - Tibial, Nerve - Tibial, Esophagus - Gastroesophageal Junction, Heart - Left Ventricle |
|  | VEGFD | Artery - Tibial, Nerve - Tibial, Esophagus - Muscularis, Thyroid, Artery - Aorta, Muscle - Skeletal |
|  | CA5BP1 | Lung |
|  | FANCB | Brain - Cortex |
| rs2271550 | PIR | Cells - Cultured fibroblasts, Thyroid, Esophagus - Muscularis, Artery - Tibial, Nerve - Tibial, Artery - Aorta, Adipose- Subcutaneous, Esophagus - Gastroesophageal Junction, Adipose - Visceral (Omentum), Brain - Caudate (basal ganglia), Brain - Nucleus accumbens (basal ganglia), Brain - Putamen (basal ganglia), Testis, Lung, Skin - Sun Exposed (Lower leg), Brain- Cortex, Skin - Not Sun Exposed (Suprapubic), Brain - Cerebellum, Muscle- Skeletal, Pituitary, Heart - Atrial Appendage, Heart- Left ventricle, Colon- Sigmoid, Brain - Cerebellar Hemisphere, Brain - Substantia nigra, Brain - Frontal Cortex (BA9), Brain- Anterior cingulate cortex (BA24), Brain - Hypothalamus, Esophagus - Mucosa, Brain- Hippocampus, Stomach, Whole blood, Breast - Mammary Tissue, Prostate, Brain - Amygdala, Colon - Transverse, Cells - EBV-transformed lymphocytes, Artery - Coronary, Ovary, Pancreas, Adrenal gland, Brain - Spinal cord (cervical c-1), Liver, Small Intestine- Terminal Ileum |
|  | VEGFD | Artery - Tibial, Nerve - Tibial, Thyroid, Muscle - Skeletal, Cells - Cultured fibroblasts, Esophagus - Muscularis, Brain - Cerebellum, Artery - Aorta, Brain - Substantia nigra, Brain - Putamen (basal ganglia), Brain - Hippocampus, Skin - Sun Exposed (Lower leg), Testis, Brain - Cortex, Brain - Frontal Cortex (BA9), Brain - Cerebellar Hemisphere, Brain - Nucleus accumbens (basal ganglia), Brain - Hypothalamus, Esophagus - Mucosa, Prostate, Brain - Anterior cingulate cortex (BA24), Brain - Caudate (basal ganglia), Pancreas, Esophagus - Gastroesophageal Junction, Heart - Atrial Appendage, Spleen |
|  | TMEM27 | Nerve - Tibial, Skin - Sun Exposed (Lower leg), Esophagus - Mucosa, Thyroid, Skin - Not Sun Exposed (Suprapubic), Brain - Cortex, Cells - Cultured fibroblasts, Artery - Aorta, Colon - Transverse, Adipose - Subcutaneous, Artery - Tibial, Brain - Cerebellum, Ovary, Brain - Nucleus accumbens (basal ganglia), Muscle - Skeletal, Heart - Left Ventricle |
|  | CA5B | Heart - Left Ventricle |
|  | CA5BP1 | Spleen |
| rs5980162 | PIR | Cells - Cultured fibroblasts, Esophagus - Muscularis, Thyroid, Nerve - Tibial, Artery - Tibial, Esophagus - Gastroesophageal Junction |
|  | VEGFD | Artery - Tibial, Nerve - Tibial |
|  | TMEM27 | Artery - Coronary, Nerve - Tibial |
| rs6629104 | PIR | Cells - Cultured fibroblasts, Adipose - Subcutaneous, Esophagus - Muscularis, Skin - Sun Exposed (Lower leg), Thyroid, Artery - Tibial, Artery - Aorta, Lung, Nerve - Tibial, Skin - Not Sun Exposed (Suprapubic), Adipose - Visceral (Omentum), Brain - Caudate (basal ganglia), Brain - Cerebellar Hemisphere, Muscle - Skeletal, Breast - Mammary Tissue, Colon- Transverse, Esophagus - Muscularis, Testis, Brain- Cerebellum, Brain - Putamen (basal ganglia), **Brain - Nucleus accumbens (basal ganglia)**, Esophagus - Gastroesophageal Junction, Brain - Cortex, Artery - Coronary, Pituitary, Brain - Hippocampus, Colon - Sigmoid, Brain - Hypothalumus, Heart - Atrial Appendage, Brain- Anterior cingulate cortex (BA24), Whole blood, Prostate, Brain - Frontal Cortex (BA9), Brain - Amygdala, Heart - Left Ventricle, Adrenal gland, Spleen, Pancreas |
|  | VEGFD | Brain - Cerebellar Hemisphere, Brain - Putamen (basal ganglia), Brain - Caudate (basal ganglia), Muscle - Skeletal, Esophagus - Mucosa, Thyroid, Skin - Sun Exposed (Lower leg), Cells - Cultured fibroblasts, Brain - Amygdala, Prostate, Spleen, Brain - Hypothalamus, Brain - Cortex |
|  | TMEM27 | Thyroid, Esophagus - Mucosa, Nerve - Tibial |
|  | CA5B | Heart - Left Ventricle |
|  | CA5BP1 | Skin - Sun Exposed (Lower leg), Lung |
|  | ACE2 | **Brain - Nucleus accumbens (basal ganglia)** |
| rs6629105 | PIR | Cells - Cultured fibroblasts, Adipose - Subcutaneous, Esophagus - Muscularis, Skin - Sun Exposed (Lower leg), Artery - Aorta, Artery - Tibial, Thyroid, Skin - Not Sun Exposed (Suprapubic), Lung, Nerve - Tibial, Adipose - Visceral (Omentum), Brain - Caudate (basal ganglia), Breast - Mammary Tissue, Muscle - Skeletal, Brain - Cerebellar Hemisphere, Esophagus - Muscularis, Colon- Transverse, Brain- Cerebellum, Testis, Brain - Putamen (basal ganglia), Esophagus - Gastroesophageal Junction, **Brain - Nucleus accumbens (basal ganglia)**, Brain - Cortex, Artery - Coronary, Brain - Hippocampus, Pituitary, Colon - Sigmoid, Brain - Anterior cingulate cortex (BA24), Heart - Atrial Appendage, Brain - Hypothalumus, Whole blood, Prostate, Brain - Frontal Cortex (BA9), Brain - Amygdala, Spleen, Heart - Left Ventricle, Pancreas |
|  | VEGFD | Brain - Cerebellar Hemisphere, Brain - Putamen (basal ganglia), Brain - Caudate (basal ganglia), Muscle - Skeletal, Cells - Cultured fibroblasts, Thyroid, Skin - Sun Exposed (Lower leg), Esophagus - Mucosa, Spleen, Brain - Cortex, Brain - Amygdala, Prostate, Brain - Hypothalamus |
|  | TMEM27 | Thyroid, Esophagus - Mucosa, Nerve - Tibial |
|  | CA5B | Heart - Left Ventricle |
|  | CA5BP1 | Skin - Sun Exposed (Lower leg) |
|  | ACE2 | **Brain - Nucleus accumbens (basal ganglia)** |
| rs113506453 | CA5BP1 | Nerve - Tibial, Testis |
