# Supplementary figures and images for: Role of the redox state of the Pirin-bound cofactor on interaction with the master regulators of inflammation and other pathways
Source: PLoS One. 2023 Nov 30;18(11):e0289158. doi: 10.1371/journal.pone.0289158 (PMC10688961; doi:10.1371/journal.pone.0289158)

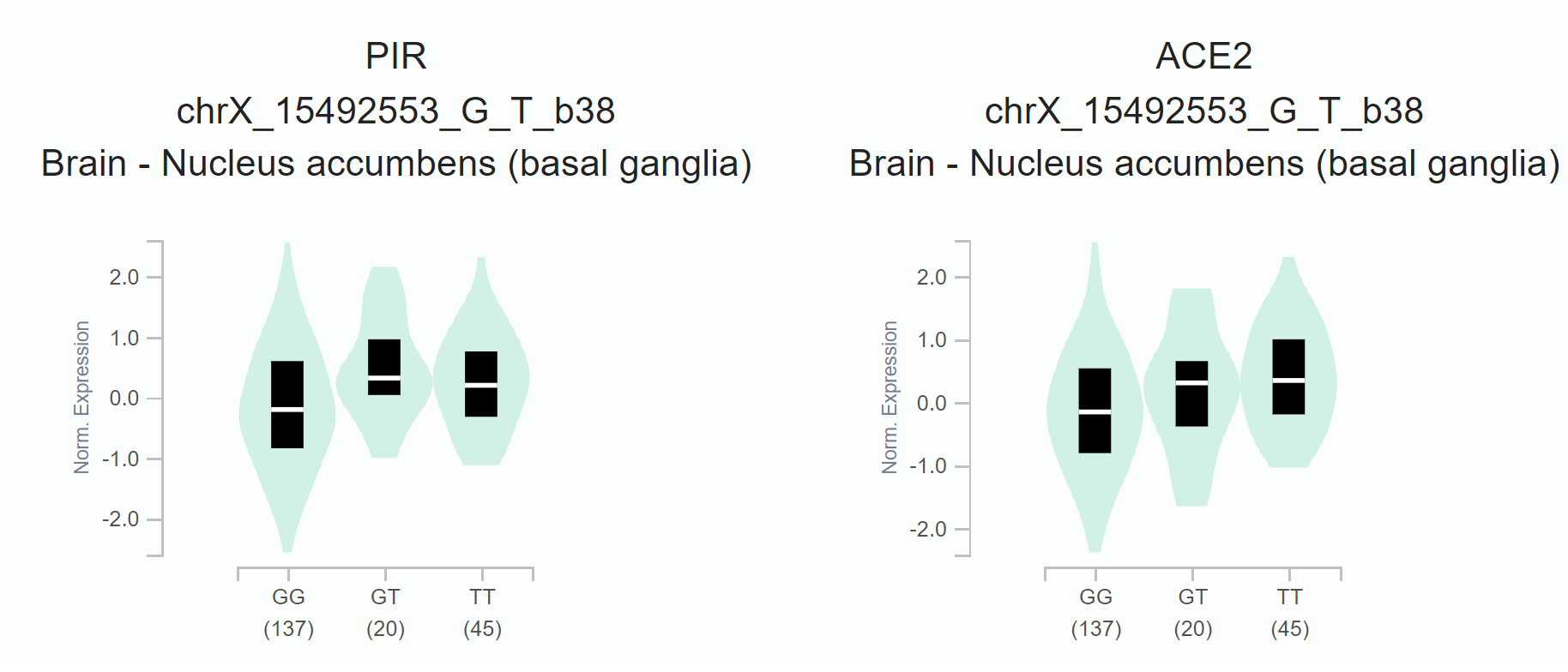

Supplement: S1 Fig — The number of subjects is shown under each genotype. The median value of the gene expression at each genotype is indicated by the white lines in the black box plots. (PNG) [file pone.0289158.s003.png]
