# Supplementary material for: Role of the redox state of the Pirin-bound cofactor on interaction with the master regulators of inflammation and other pathways
Source: PLoS One. 2023 Nov 30;18(11):e0289158. doi: 10.1371/journal.pone.0289158 (PMC10688961; doi:10.1371/journal.pone.0289158)
Supplement: Attachment — Submitted filename: Response to Reviewers.doc [file pone.0289158.s004.doc]

**Response to academic editor and reviewer**

**Manuscript #** PONE-D-23-03196

**Manuscript title:** Role of the redox state of the Pirin-bound cofactor on interaction with the master regulators of inflammation and other pathways

**Responses to the academic editor**

1. Please ensure that your manuscript meets PLOS ONE's style requirements, including those for file naming. The PLOS ONE style templates can be found at

<https://journals.plos.org/plosone/s/file?id=wjVg/PLOSOne_formatting_sample_main_body.pdf>  and

<https://journals.plos.org/plosone/s/file?id=ba62/PLOSOne_formatting_sample_title_authors_affiliations.pdf>

**Response:** We have updated the manuscript following PLOS ONE's style requirements.

2. Thank you for stating the following in the Acknowledgments Section of your manuscript:

"This study was supported by a grant under the Special Allocation in Science and Technology from the Ministry of Science and Technology, Bangladesh to AAS. The authors are thankful for the support."

We note that you have provided additional information within the Acknowledgements Section that is not currently declared in your Funding Statement. Please note that funding information should not appear in the Acknowledgments section or other areas of your manuscript. We will only publish funding information present in the Funding Statement section of the online submission form.

Please remove any funding-related text from the manuscript and let us know how you would like to update your Funding Statement. Currently, your Funding Statement reads as follows:

"This study was supported by a grant under the Special Allocation in Science and Technology from the Ministry of Science and Technology, Bangladesh"

Please include your amended statements within your cover letter; we will change the online submission form on your behalf.

**Response:** We have removed the funding-related information from the Acknowledgements Section. The current Funding Statement is OK.

3. Please ensure that you include a title page within your main document. We do appreciate that you have a title page document uploaded as a separate file, however, as per our author guidelines ([http://journals.plos.org/plosone/s/submission-guidelines#loc-title-page](http://journals.plos.org/plosone/s/submission-guidelines#loc-title-page)) we do require this to be part of the manuscript file itself and not uploaded separately.

Could you therefore please include the title page into the beginning of your manuscript file itself, listing all authors and affiliations.

**Response:** We have updated the title page according to the guidelines. It is now included at the beginning of the main manuscript.

4. Thank you for stating the following financial disclosure:

"This study was supported by a grant under the Special Allocation in Science and Technology from the Ministry of Science and Technology, Bangladesh."

Please state what role the funders took in the study.  If the funders had no role, please state: "The funders had no role in study design, data collection and analysis, decision to publish, or preparation of the manuscript."

If this statement is not correct you must amend it as needed.

Please include this amended Role of Funder statement in your cover letter; we will change the online submission form on your behalf.

**Response:** We could not find the section for “Financial Disclosure” on the “Additional Information” page. We would like to mention the roles of the funders in the revised manuscript as "This study was supported by a grant under the Special Allocation in Science and Technology from the Ministry of Science and Technology, Bangladesh. The funders had no role in study design, data collection and analysis, decision to publish, or preparation of the manuscript".

5. Please review your reference list to ensure that it is complete and correct. If you have cited papers that have been retracted, please include the rationale for doing so in the manuscript text, or remove these references and replace them with relevant current references. Any changes to the reference list should be mentioned in the rebuttal letter that accompanies your revised manuscript. If you need to cite a retracted article, indicate the article’s retracted status in the References list and also include a citation and full reference for the retraction notice.

**Response:** We have reviewed the reference list following the suggestions.

**Responses to the reviewer**

1. The information regarding different diseases and their possible cure mentioned in the article is well explained so I am going to recommend this article for publication. it's very well written. Only the future perspectives for further research in this specific aspect should also mentioned.

**Response:** Thank you for your remarks on the manuscript. We have mentioned the future perspectives for future research in the revised manuscript (highlighted in yellow).
